# Severe and Persistent Depletion of Circulating Plasmacytoid Dendritic Cells in Patients with 2009 Pandemic H1N1 Infection

**DOI:** 10.1371/journal.pone.0019872

**Published:** 2011-05-19

**Authors:** Miriam Lichtner, Claudio M. Mastroianni, Raffaella Rossi, Gianluca Russo, Valeria Belvisi, Raffaella Marocco, Claudia Mascia, Cosmo Del Borgo, Fabio Mengoni, Ilaria Sauzullo, Gabriella d'Ettorre, Claudia D'Agostino, Anna P. Massetti, Vincenzo Vullo

**Affiliations:** 1 Infectious Diseases Unit, “Sapienza” University, Polo Pontino, SM Goretti Hospital, Latina, Italy; 2 Department of Public Health and Infectious Diseases, Istituto Pasteur-Fondazione Cenci-Bolognetti, “Sapienza” University, Rome, Italy; Albany Medical College, United States of America

## Abstract

**Background:**

Dysregulation of host immune responses plays a critical role in the pathogenesis of severe 2009 pandemic H1N1 infection. Whether H1N1 virus could escape innate immune defense in vivo remains to be investigated. The aim of this study was to evaluate the pattern of innate immune response during human 2009 H1N1 infection. We performed the enumeration of circulating myeloid dendritic cells (mDC) and plasmacytoid DC (pDC) in blood from patients with H1N1 pneumonia shortly after the onset of symptoms and during follow-up at different intervals of time. The analysis of CD4 and CD8 count, CD38 T-cell activation marker and serum cytokine/chemokine plasma levels was also done.

**Methodology/Principal Findings:**

Blood samples were collected from 13 hospitalized patients with confirmed H1N1-related pneumonia at time of admission and at weeks 1, 4, and 16 of follow-up. 13 healthy donors were enrolled as controls. In the acute phase of the disease, H1N1-infected patients exhibited a significant depletion in both circulating pDC and mDC in conjunction with a decrease of CD4 and CD8 T cell count. In addition, we found plasmatic hyperproduction of IP-10 and RANTES, whereas increase in T-cell immune activation was found at all time points. When we assessed the changes in DC count over time, we observed a progressive normalization of mDC number. On the contrary, H1N1-infected patients did not achieve a complete recovery of pDC count as values remained lower than healthy controls even after 16 weeks of follow-up.

**Conclusions:**

H1N1 disease is associated with a profound depletion of DC subsets. The persistence of pDC deficit for several weeks after disease recovery could be due to H1N1 virus itself or to a preexisting impairment of innate immunity.

## Introduction

Since the onset of the 2009 influenza A (H1N1) pandemic, the virus has caused significant morbidity and mortality [1]. Although older patients and those with comorbidities are more likely to experience worse clinical outcomes, many severe cases occur in healthy young adults; approximately one quarter to one half of patients with 2009 H1N1 virus infection who were hospitalized or died had no reported coexisting medical conditions [2]–[4]. The pattern of host immune response to 2009 H1N1 virus infection in humans is not fully elucidated [5]. Recent studies have showed that increased cytokine and chemokine plasma levels are markers of critical illness, suggesting that immunopathology may contribute to the disease severity [6], [7]. An important role of Th1 and Th17 response in H1N1 infection as early distinctive hallmarks of severe respiratory impairment has also been suggested [8]. In addition, T cells from pandemic H1N1-infected patients presenting with severe or fatal clinical course resulted in impaired effector cell differentiation and profound functional anergy [9]. To date, the role of host innate immune responses in H1N1-infected patients has been little explored. In a recent study assessing the effect of 2009 H1N1 virus on the human immune system, it has been shown that infection by the H1N1 virus is accompanied by a characteristic impairment of the innate immune responses characterized by a selective defect in cytokine responses to *Streptococcus pneumoniae* [10]. Osterlund *et al.* also showed that the 2009 H1N1 virus was able to escape the host innate immune response by interfering with antiviral and proinflammatory cytokine gene expression in human macrophages and dendritic cells (DC) [11].

DC are key effector cells in innate immunity for the first line defense against viral infection [12]–[14]. During influenza virus infection, DC secrete antiviral cytokines, activate virus-destroying natural killer cells and stimulate T cells with production of neutralizing antibodies, which, together, are responsible for virus clearance. Two types of DC that can be identified by phenotypic markers and function are circulating in immature form in human blood: myeloid DC (mDC) and plasmacytoid DC (pDC). mDC express CD11c, require granulocyte-macrophage colony-stimulating factor (GM-CSF) for growth and survival and perform antigen uptake, T cell activation and secretion of interleukin (IL)-12 and IL-18. pDC express CD123, are dependent on IL-3 for survival and rapidly produce large amounts of interferon (IFN)-α type I in response to viral infections, including influenza virus. IFN-α production by pDC plays a pivotal role in immune response against flu [15].

In order to investigate the pattern of innate immune response during human 2009 H1N1 virus infection, we performed a longitudinal assessment of DC in blood from patients with H1N1 pneumonia shortly after the onset of symptoms and during follow-up at different intervals of time. A direct enumeration of circulating mDC and pDC was performed in conjunction with analysis of CD4 and CD8 count, T-cell activation markers and serum cytokine/chemokine plasma levels. Our data showed that 2009 pandemic H1N1 virus infection is associated with a severe impairment of the innate immune responses characterized by a profound depletion of pDC subset, which persists for several weeks after recovery of the disease.

## Materials and methods

### Ethics Statement

The study was approved by the institutional review board (Department of Infectious and Tropical Diseases, Sapienza University of Rome). All study participants gave informed written consent.

### Study population

The study population included a total of 13 patients with confirmed 2009 H1N1 influenza A admitted at the Infectious Disease Units of Sapienza University of Rome (Policlinico Umberto I Hospital) and Latina (SM Goretti Hospital). Nasopharyngeal-swab specimens were collected at admission and real-time reverse-transcriptase polymerase chain reaction (RT-PCR) was performed in each institution or centralized in a reference laboratory. All enrolled patients had acute respiratory illness with opacities found on the chest X-ray (revealing pneumonia) and had laboratory-confirmed pandemic 2009 H1N1 virus infection by real-time RT-PCR. No microbiologic evidence of other viral or bacterial respiratory infections was detected. 13 sex and aged-matched healthy controls with no flu symptoms in the last 4 weeks were enrolled as controls. For each enrolled patients venous blood samples were collected into heparinized glass tubes and ethylenediaminetetraacetic acid (EDTA)-containing tubes at time of admission (week 0) and at week 1, 4, and 16 of follow-up.

### Reagents

TruCOUNT™ tubes, FACS Lysing Solution, and monoclonal antibodies (mAbs) for DC labeling anti-CD45- peridinin chlorophyll (PerCP), anti-HLA-DR- allophycocyanin (APC), Lineage-fluorescein isothiocyanate (FITC) cocktail composed of anti-CD3 (SK7), anti-CD14 (MP9), anti-CD16 (3G8), anti-CD19 (SJ25C1), anti-CD20 (L27), anti-CD56 (NCAM 16-2), and anti-CD11c- phycoerythrin (PE) or anti-CD123-PE), for isotype control (mouse anti-IgG1a-PE and mouse anti-IgG2a-PE) and for CD4 count CD4-FITC and CD8-PerCP were used. All mAbs, TruCOUNT™ tubes and FACS Lysing Solution were purchased from Becton Dickinson (BD Biosciences Pharmingen, Italy).

### Enumeration of plasmacytoid DC and myeloid DC

DC enumeration was done using TruCOUNT tubes and lyse no-wash whole blood analysis, as previously described [16], [17]. Samples were analyzed within 1–3 h of staining using a FACScalibur flow cytometer and CellQuest version *1.0* (Becton Dickinson, Mountain View CA). All data were collected using identical instrument settings. DC were identified as lineage-negative cells and HLA-DR-APC positive cells. Then to define mDC and pDC, CD11c-PE and CD123-PE were respectively used in two different tubes. To calculate the blood absolute pDC or mDC number (cells/ml) we used the known formula as previously described [16], [17].

### Monocyte and CD4 and CD8 T-cell count

A standard whole blood lyse/no wash TruCOUNT assay was used to calculate also the number of T lymphocytes CD4+ and CD8+ using anti-CD4-PE and anti-CD8-FITC and anti-CD45-PerCP. Monocytes were identified with anti-CD14-PE and anti-CD45-PerCP.

### Measurement of CD4 and CD8 T-cell activation

CD4 and CD8 cell activation was assessed on whole blood cells by using a lyse/no wash procedure. The percentage of CD4 and CD8 cells that expressed CD38 and human leukocyte antigen (HLA)-DR, activation markers, was determined using PE-conjugated anti-CD38, FITC-conjugated anti-HLA-DR, PerCP-coniugated anti-CD45- and APC-conjugated anti-CD4, or APC-conjugated anti-CD8 monoclonal antibodies and analyzed by flow cytometry.

### Cytokine and chemokine determination

Cytokines and chemokines were measured in plasma prepared from blood that had been collected into EDTA-containing tubes and stored at −80°C. Instant enzyme-linked immunosorbent assays (ELISA) (Bender MedSystems, Vienna, Austria) were used for the quantitative detection of IFN-α, tumour necrosis factor (TNF)- α, IL-6, CCL3/macrophage inflammatory protein-1 (MIP-1α), CCL4/macrophage inflammatory protein-1 (MIP-1β), CCL5/regulated upon activation, normal T-cell expressed, and secreted (RANTES). Quantikine ELISA (R&D system, Abingdon, UK) was used for testing IFN-γ-inducible protein 10 (IP-10), IFN-γ, IL-15 and IL-17. The assays were performed in accordance with the instructions of the manufacturers. The detection limit of each cytokine assay was: 3.16 pg/ml for IFN-α, 1.65 pg/ml for TNF-α, 8 pg/ml for IFN-γ, 2 pg/ml for IL-15, 15 pg/ml for IL-17, 1.67 pg/ml for IP-10, 0.92 pg/ml for IL-6, 6 pg/ml for CCL3/MIP-1α, 3 pg/ml for CCL4/MIP-1β, 4.2 pg/ml for CCL5/RANTES. For statistical analysis, samples below the detection limit were arbitrarily assigned the value for the detection limit in the assays.

### Statistical analysis

SPSS version 13.0 for windows (SPSS Inc., *Apache Software Foundation*, Chicago, Illinois) was used. Results are presented as median and range. Data at multiple time points were compared using the Friedman two-way analysis of variance by ranks followed by the Dunnett's multiple comparison post-test. The differences of values between the healthy controls and the patients were analyzed using the non-parametric Mann-Whitney U-test. The significance of correlation analysis was estimated using the Spearman's rank correlation test. P-value <0.05 was regarded as significant.

## Results

### Demographics and clinical characteristics of study population

Among the 13 enrolled patients, six were males and seven females (median age, 44 years; range 31–56) (Table 1). None of the patients had received the vaccine against pandemic 2009 H1N1 virus. Two patients were pregnant, 7 had obesity with a body mass index (BMI) >30, 3 had chronic obstructive pulmonary disease (COPD); 1 was diabetic; 5 subjects didn't have any known risk factor. The median days from onset of symptoms to hospitalization was 3 (range 1–4). All patients received oseltamivir by the day of hospitalization. Lung involvement with more than one opacity in initial x-ray chest was present in 11 of 13 patients. Eight patients showed a PaO_2_/FiO_2_ ratio below 250 assessed at 0.31% of pO_2_. Three patients required non-invasive ventilation by continuous positive airway pressure (CPAP). No patients required mechanical ventilation.

**Table 1:** Demographic and clinical characteristics of the 13 patients affected by pandemic H1N1 influenza.

Patient code	Age (years)	Sex	Risk factors	BMI	O_2_ Sat (%)	PaO_2_/FiO_2_ ratio	Ventilatory support	Quadrant lung involvement (No)
001	48	F	Obesity/Type II diabetes	49	72	175	CPAP	4
002	51	M	Obesity	35	84	207	CPAP	3
003	31	M	Obesity/COPD	58	82	309	O_2_ therapy by ventimask	4
004	53	M	Obesity/COPD	34	79	214	CPAP	3
005	31	F	None	20	95	309	O_2_ therapy by ventimask	2
006	30	F	Pregnancy	25	98	428	None	1
007	38	F	None	24	94	350	O_2_ therapy by ventimask	2
008	52	M	Obesity/Asthma	38	91	313	O_2_ therapy by ventimask	4
009	38	M	None	27	90	250	O_2_ therapy by ventimask	1
010	44	M	Obesity	34	85%	302	O_2_ therapy by ventimask	2
011	31	F	None	27	89	299	O_2_ therapy by ventimask	2
012	56	M	COPD/Obesity	32	77	245	CPAP	3
013	45	F	None	23	82	289	O_2_ therapy by ventimask	2

BMI: body mass index; Sat: saturation; CPAP: continuous positive airway pressure; COPD: chronic obstructive pulmonary disease.

### Circulating plasmacytoid DC and myeloid DC

**Figure 1:**
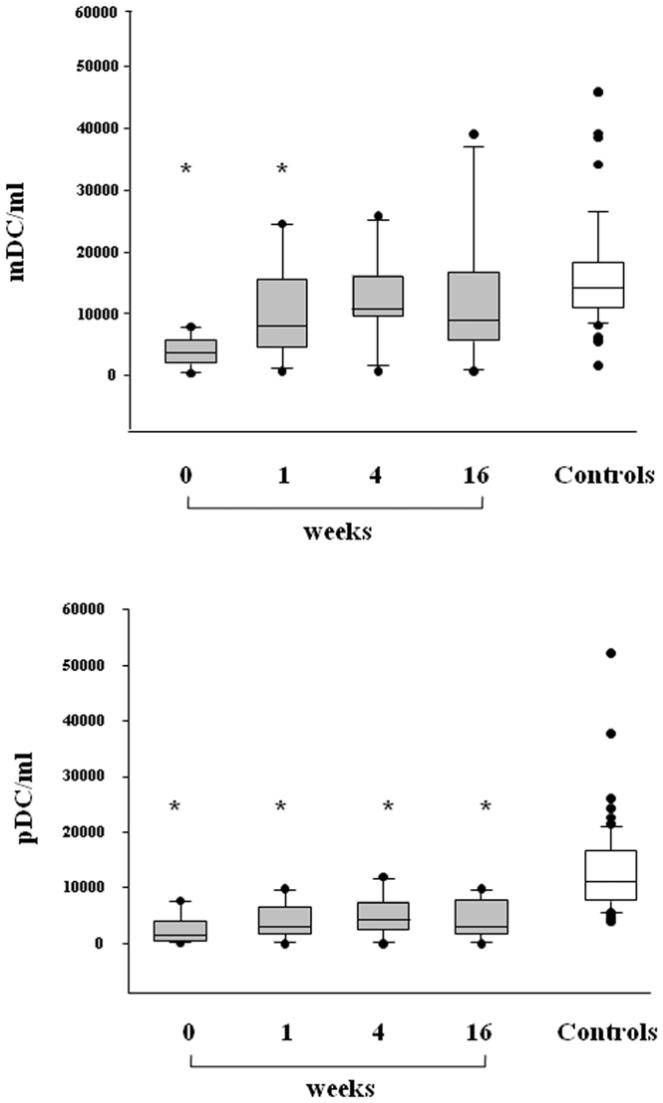
Circulating myeloid dendritic cells (mDC) and plasmacytoid DC (pDC) in pandemic H1N1-infected patients and controls. Box plots show the 10th, 25th, 50th(median), 75th and 90th percentile and outlying values. Gray box plots represent the number of circulating mDCs (upper panel) and pDCs (lower panel) in 13 H1N1-infected patients at baseline (week 0) and during follow-up (week 1, 4 and 16). White box plots indicate 13 sex and aged-matched healthy controls. Asterisks indicate significant decrease of mDC and pDC (p<0,05 versus controls).

**Figure 2:**
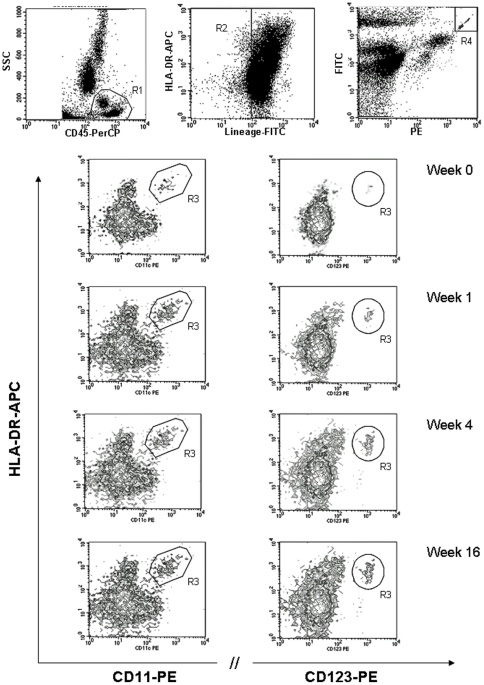
Cytometry analysis of mDC and pDC in one representative H1N1-infected patient at baseline and during follow-up. The gating strategy to define DC population (lineage negative, HLA-DR positive) is illustrated in the upper dot plots. In the lower contour plots mDC (CD11c+) and pDC (CD123+) definition is showed at diagnosis (week 0) and during follow-up (week 1, 4 and 16).

Circulating pDC and mDC were quantified in 13 patients with H1N1 infection at the time of hospitalization and at different intervals of time during follow-up using the single-platform TruCOUNT assay (Figure 1). A significant reduction in both circulating pDC and mDC was found in H1N1-infected patients at baseline when compared with 13 age- and sex-marched healthy donors (p<0.001 for both). Indeed, in patients affected by 2009 pandemic H1N1 influenza the median (range) DC counts (cells/ml) were as follows: 1420 (range 7655–88) for pDC versus 10967 (3875–52111) of controls and and 3652 (7897–387) for mDC versus 14064 (1662–45917) of controls. During follow-up, we observed a rapid and significant augmentation in mDC numbers; indeed, the median (range) mDC count increased to 8034 (720–24603) at week 1, 10700 (742–25781) at weeks 4, and 8986 (750–39057) at week 16 (p< 0.05 for weeks 1, 4, and 16 versus baseline). At the end of follow-up the values of mDC were within normal ranges. With regard to pDC, there was a slight increase of the cells over time; the median of pDC increased to 2882 (0–9856) at week 1, 4287 (26–11985) at weeks 4, and 3013 (32–9805) at week 16 (p <0.05 for weeks 4, and 16 versus baseline). Interestingly, H1N1-infected patients did not achieve a complete recovery of pDC count as values remained significantly lower than healthy controls at all time points. Although obesity was considered a major risk factor for the severity of lung disease, no correlation between DC count and BMI was found. A representative cytometry analysis of one H1N1-infected patient during follow-up is showed in figure 2. During the current seasonal flu we have examined 4 outpatients with mild H1N1 disease (no pulmonary involvement) and no deficit in the peripheral numbers of either mDC or pDC was found (data not shown).

### Circulating monocytes, CD4+ and CD8+ T-cell count

Changes in monocytes, CD4+ and CD8+ T-cells in study population are shown in Table 2. Values of monocytes were within normal ranges in H1N1-infected patients and no significant changes were found over time in comparison to controls. On the other hand, baseline CD8 and CD4 T lymphocyte count was significantly lower compared with healthy controls (p<0.05 for both). At follow-up analysis, we observed a prompt and significant increase in both CD4 and CD8 count with normalization of values.

**Table 2:** Blood monocytes, CD4+ and CD8+ T-cell count in 13 patients with pandemic H1N1 influenza and 13 healthy controls.

	Patients				Controls
	Week 0	Week 1	Week 4	Week 16	
**Monocytes cells/ml**	542 (187–1202)	612 (212–1248)	597 (235–1210)	420 (154–1002)	560 (421–1006)
**CD4 + T cells/mmc**	*360 (112–1057)	573 (215–1371)	835 (248–1458)	875 (321–1622)	888 (758 1521)
**CD8 + T cells/mmc**	*268 (93–544)	388 (151–835)	525 (168–903)	517 (187–900)	558 (325–908)

Results are expressed in median (range).

Asterisks indicate significant decrease (p<0.05 versus controls).

### T-cell activation markers

**Figure 3:**
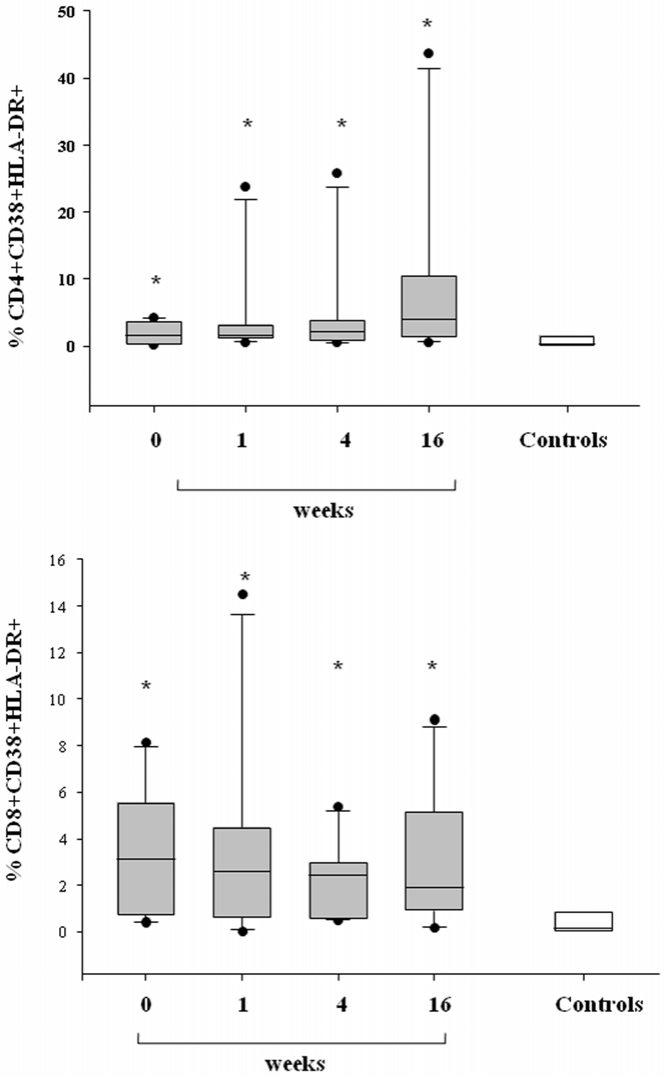
CD4+ and CD8+ T cell activation markers in pandemic H1N1-infected patients and controls. Box plots show the 10^th^, 25^th^, 50^th^ (median), 75^th^ and 90^th^ percentile and outlying values. Gray box plots represent the percentage of CD4+ T cells (upper panel) and CD8+ T cells (lower panel) that express activation markers CD38/HLA-DR in 13 H1N1-infected patients at baseline (week 0) and during follow-up (week 1, 4, and 16). White box plots indicate 13 sex and aged-matched healthy controls. Asterisks indicate a significant increase in percentage of double positive DR+/CD38+ in both CD4+ and CD8+ T cells (p<0,05 versus controls).

In order to measure the degree of T-cell activation during 2009 pandemic H1N1 virus infection, we assessed the percentage of CD4+ and CD8+ cells that expressed the activation markers CD38/HLA-DR (Figure 3). Results showed H1N1-infected patients exhibited at baseline a significantly higher percentage of double positive DR+/CD38+ in both CD4 and CD8 in comparison with controls (median [range] 1,65 [0.25–4.24] for DR+/CD38+/CD4+ and 3,1 [0.42–8.14] for DR+/CD38+/CD8+ versus 0.3% [0.013–2.12] and 0.15% [0.00–3.2] of controls, respectively) (p<0.05). T-cell immune activation markers persisted elevated during the overall period with a significant difference between cases and controls at week 16 of follow-up (p<0.01).

### Plasma concentrations of cytokines and chemokines

**Figure 4:**
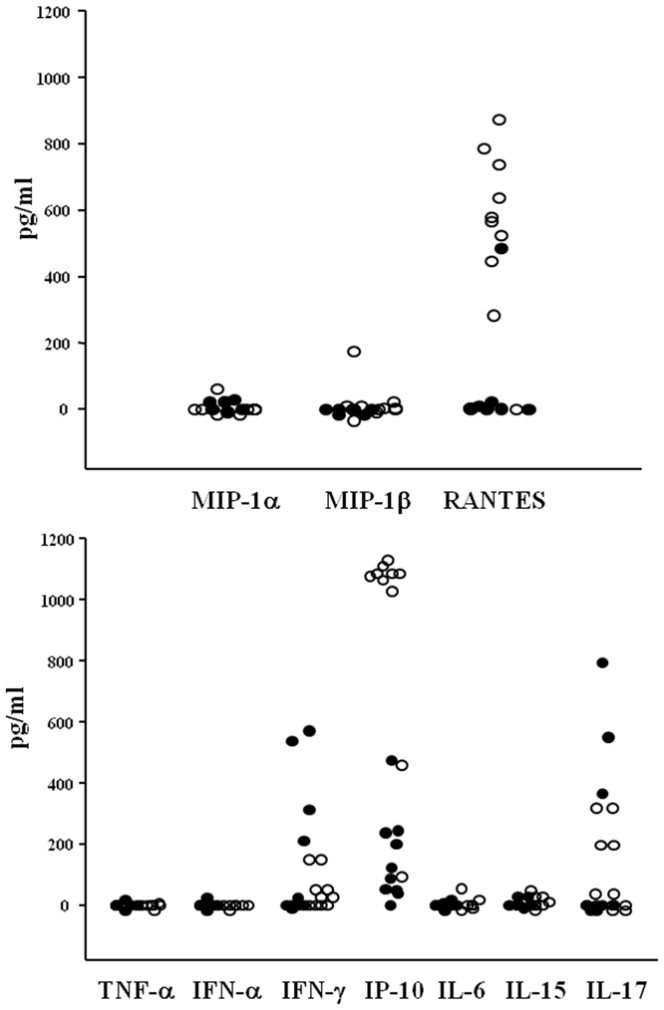
Plasma levels of chemokines and cytokines in pandemic H1N1-infected patients and controls. Dot plot represent circulating levels of chemokines CCL3/MIP-1α, CCL4/MIP-1β, CCL5/RANTES (upper panel) and cytokines TNF-α, IFN-α, IFN-γ, IP-10, IL-6, IL-15, IL-17 (lower panel) in 13 H1N1-infected patients at the time of hospitalization. Black circles indicate 13 sex and aged-matched healthy controls. Only the levels of IP-10 and RANTES were significantly higher in patients in comparison with the control group (p<0.01 for both).

In another set of experiments, we assessed circulating levels of cytokines (TNF-α, IFN-α, IFN-γ, IP-10, IL-6, IL-15, IL-17) and chemokines CCL3/MIP-1α, CCL4/MIP-1β, CCL5/RANTES (Figure 4). No detectable plasma concentrations of TNF-α, IL-6, IL-15 and IFN-α were found. CCL3/MIP-1α, CCL4/MIP-1β, IFN-γ and IL-17 were detectable in small amounts in a low number of patients. On the other hand, the highest concentrations were found for IP-10 (median 1079 pg/ml, range 92-1137) and RANTES (median 647 pg/ml, range 0–875). These levels were significantly higher than those obtained in the control group (p<0.01 for both IP-10 and RANTES) (Figure 4). Longitudinal analysis showed a rapid decrease of altered cytokine IP-10 and chemokine RANTES just after 1 week of follow-up (data not shown).

## Discussion

The novel 2009 H1N1 virus infection emerged as the first influenza pandemic of the 21^st^ century and it has been associated with acute severe pneumonia that resulted in high morbidity and mortality [18], [19]. Although smoking, pregnancy and obesity have been highlighted as risk factors for severe pneumonia, high percentage of fatal cases had no evident underlying medical condition and occurred in previously healthy individuals [2]. Whether the severity of human 2009 H1N1 disease could be due to the direct pathogenic effect of virus itself or to a dysregulation of the immune system remains to be investigated. Recent lines of evidence suggest that a virus-driven dysregulation of host response leading to an exaggerated production of inflammatory cytokines and chemokines plays a contributing role in the pathogenesis and severity of human H1N1 disease [8].

In the present study, we have investigated the role of innate immune response in patients with H1N1 2009 related-pneumonia by assessing in the peripheral blood the circulating number of DC. We demonstrated that during the acute phase H1N1-infected patients exhibited a specific and significant decrease in the absolute number of circulating DC when compared to healthy donors. The reduction affects the two main subsets of DC circulating in the peripheral blood, i.e. mDC and pDC. The importance of DC in the initiation and control of innate and adaptive immune responses against influenza infection is well documented [14]–[16]. Once activated, DC promote the stimulation of CD4 and CD8 T cells by inducing a specific response with the production of neutralizing antibodies and secretion of direct antiviral cytokines, such as IFN-α, which, together, contribute to elimination of viral infection. The different subpopulations of DC are involved in the regulatory immune mechanisms during influenza. mDC are characterized by a high susceptibility to influenza virus and, when infected, produce cytokines, as IL-12, IL-23 and TNF-α, and may induce the activation of specific cytotoxic CD8+ T lymphocytes via IL-15. pDC are the main IFN-α producing cells and are essential for viral clearance [20]. In our patients the levels of circulating IFN-α were persistently undetectable. Whether the impairment in IFN-α secretion could be related to a clear defect in the function of pDC or to a simple numerical reduction of these fundamental antigen presenting cells (APC) is unclear. Various possible mechanisms could be involved in peripheral DC deficiency during H1N1 disease. One hypothesis is that these cells are more susceptible to apoptosis during viral infection and may undergo peripheral destruction. However, it is conceivable that the low number of peripheral DC fractions could be due to their enhanced recirculation from blood to affected tissues, such as inflamed lung or lymph nodes. Recent experiments in mouse showed that a subset of DC, described as TNF-α inducible nitric oxide synthase (iNOS)-producing DC (tipDC) [21], accumulate in significantly greater numbers in the lung during the course of lethal influenza [22]. The DC recirculation has also been described in HIV infection where the deficit of pDC has been associated to an augmentation in lymph nodes and spleen [23], [24].

In our patients in the acute phase of disease we also found a significant decrease of CD4 and CD8 T cells, while monocytes were within normal ranges. In addition, at the time of admission, we observed an increase in CD38 T-cell immune activation and plasmatic hyperproduction of IP-10 and RANTES. These findings are in agreement with a recent study which demonstrated that the early immune response in H1N1-infected patients is characterized by a rapid generalized lymphopenia associated with preferential loss of Th17 population and marked T cell activation [25]. The deficit of T-cell count was promptly restored just after 1 week of follow-up in conjunction with a rapid decrease of cytokine IP-10 and chemokine RANTES. On the other hand, the expression of T-cell CD38 immune activation marker persisted elevated during the overall period of the study. When we assessed the longitudinal changes in DC count, we observed a progressive augmentation in the number of mDC which reached normal values at the end of follow-up. On the contrary, H1N1-infected patients did not achieve a complete recovery of pDC count as values remained significantly lower than healthy controls during the overall period of the follow-up. During the 4 months of follow-up the persistent pDC numerical deficit was present in association with a prolonged T-cell immune activation. In summary, it was found that DCs and T cells are diminished in the blood, and T cells display an activated phenotype for the duration of the study. One interpretation of the data is that both populations of cells are recruited to other tissues, such as lymph nodes, where the cells interact and the T cells are activated.

Various possible mechanisms could be involved in the persistence of pDC depletion for several weeks even after H1N1 disease recovery. One hypothesis is that H1N1 virus itself might continue to induce a direct effect on pDC with a prolonged recirculation in the lung and lymph nodes which could lead to immune activation status. Another possible explanation could be a preexisting impairment of innate immunity. Indeed, we cannot exclude that the deficit of pDC was present in our patients before H1N1 infection and it might be responsible for viral lung spread by decreasing IFN-α production and increasing T cell activation. Recently, a close association between impaired host immune competence and a severe or fatal clinical course of H1N1 infection has been reported (9). Agrati *et al.* showed that T cells from patients with severe H1N1 infection exhibited impaired effector cell differentiation and failed to respond to mitogenic stimulation. The anergy of T-cell response is probably related to apoptotic mechanisms and could expose the host to viral damage and to secondary infections in the lung. In a recent study, H1N1-infected patients with severe respiratory disease are characterized by the impaired activation of genes participating in the development of the antiviral adaptive response and by persistence of the virus in the lung. In particular, poor expression of a number of genes involved in B-cell development, T-helper cell differentiation, CD28, granzyme B signaling, apoptosis and protein ubiquitination has been reported. In addition, the impaired expression of genes participating in dendritic cell maturation, such as CCR7, CD1C, IL-18, suggests the presence of a defective antigen presentation [26]. In the present investigation, we did not perform the analysis of gene expression profiles; however, it is possible that the persistent impairment of pDC seen in our patients could be due to altered expression levels of the genes participating in the antigen presentation pathway induced by the virus. In a large comprehensive immunologic analysis of Indian H1N1-infected patients, Arankalle *et al.* provided evidence of a profound impairment of host immune response; they showed that the majority of the immune-function genes were down-regulated in the periphery and up-regulated in the lung cells [27]. This profile of gene expression supports our hypothesis on the infiltration of monocytes/DCs/macrophages to the target pulmonary tissue for mounting immune response and/or tissue repair.

Finally, we cannot exclude that underlying conditions such as obesity, type II diabetes, asthma, or COPD could affect circulating DC count or levels or cytokines/chemokines. In particular, obesity represents major risk factor for the severity of lung disease but was present only in 7 patients. Nevertheless, we did not find any significant correlation between DC count, chemokine/cytokine levels and BMI.

Our study has obvious limitations due the lack of analysis of viral load kinetics and their potential correlation with immunologic parameters. In previous studies, a clear correlation between viral load and clinical and immunologic outcome has not been established thus suggesting that factors other than viral replication can be responsible for more severe clinical disease [9]. The present work included only subjects with confirmed influenza pneumonia; in this respect, we were not able to detect immunologic differences between different clinical patterns of H1N1 infection, ranging from asymptomatic infection to more severe form of the disease. During the current seasonal flu we are following outpatients with mild H1N1 disease (no pulmonary involvement) and until now we have not found a defect in blood number of both mDC and pDC. Unlike other studies, ours represents the first attempt to perform a longitudinal analysis of the pattern of innate immune response following several weeks after H1N1 infection.

In conclusion, the present study suggests that 2009 human H1N1 disease was associated with a profound depletion of DC subsets. The underlying mechanisms involved in the persistence of pDC deficit even after several weeks after disease recovery are not still elucidated. Nevertheless, these findings underline the importance for monitoring host innate response to H1N1 infection in order to identify not only patients who have an enhanced risk of developing severe complications, but also to detect preexisting immune impairment in apparently immunocompetent cases.

## References

[1] Bautista E, Chotpitayasunondh T, Gao Z, Harper SA, Shaw M (2010). Clinical aspects of pandemic 2009 influenza A (H1N1) virus infection.. N Engl J Med.

[2] Louie JK, Acosta M, Winter K, Jean C, Gavali S (2009). Factors associated with death or hospitalization due to pandemic 2009 influenza A (H1N1) infection in California.. JAMA.

[3] Dominguez-Cherit G, Lapinsky SE, Macias AE, Pinto R, Espinosa-Perez L (2009). Critically ill patients with 2009 influenza A(H1N1) in Mexico.. JAMA.

[4] Kumar A, Zarychanski R, Pinto R, Cook DJ, Marshall J (2009). Critically ill patients with 2009 influenza A (H1N1) infection in Canada.. JAMA.

[5] Peiris JS, Hui KP, Yen HL (2010). Host response to influenza virus: protection versus immunopathology.. Curr Opin Immunol.

[6] To KK, Hung IF, Li IW, Lee KL, Koo CK (2010). Delayed clearance of viral load and marked cytokine activation in severe cases of pandemic H1N1 2009 influenza virus infection.. Clin Infect Dis.

[7] Hagau N, Slavcovici A, Gonganau DN, Oltean S, Dirzu DS (2010). Clinical aspects and cytokines response in severe H1N1 influenza A virus infection.. Crit Care.

[8] Bermejo-Martin JF, Ortiz de Lejarazu R, Pumarola T, Rello J, Almansa R (2009). Th1 and Th17 hypercytokinemia as early host response signature in severe pandemic influenza.. Crit Care.

[9] Agrati C, Gioia C, Lalle E, Cimini E, Castilletti C (2010). Association of profoundly impaired immune competence in H1N1v-infected patients with a severe or fatal clinical course.. J Infect Dis.

[10] Giamarellos-Bourboulis EJ, Raftogiannis M, Antonopoulou A, Baziaka F, Koutoukas P (2009). Effect of the novel influenza A (H1N1) virus in the human immune system.. PloS One.

[11] Osterlund P, Pirhonen J, Ikonen N, Rönkkö E, Strengell M (2009). Pandemic H1N1 2009 influenza A virus induces weak cytokine responses in human macrophages and dendritic cells and is highly sensitive to the antiviral actions of interferons.. J Virol.

[12] Banchereau J, Steinman RM (1998). Dendritic cells and the control of immunity.. Nature.

[13] Hao X, Kim TS, Braciale TJ (2008). Differential response of respiratory dendritic cell subsets to influenza virus infection.. J Virol.

[14] Liu WC, Lin SC, Yu YL, Chu CL, Wu SC (2010). Dendritic cell activation by recombinant hemagglutinin proteins of H1N1 and H5N1 influenza A viruses.. J Virol.

[15] Wolf AI, Buehler D, Hensley SE, Cavanagh LL, Wherry EJ (2010). Plasmacytoid dendritic cells are dispensable during primary influenza virus infection.. J Infect Dis.

[16] Lichtner M, Rossi R, Rizza C, Mengoni F, Sauzullo I (2008). Plasmacytoid dendritic cells count in antiretroviral-treated patients is predictive of HIV load control independent of CD4+ T-cell count.. Curr HIV Res.

[17] Hosmalin A, Lichtner M, Louis S (2008). Clinical analysis of dendritic cell subsets: the dendritogram.. Methods Mol Biol.

[18] Fowlkes AL, Arguin P, Biggerstaff MS, Gindler J, Blau D (2011). Epidemiology of 2009 Pandemic Influenza A (H1N1) Deaths in the United States, April–July 2009.. Clin Infect Dis.

[19] Bautista E, Chotpitayasunondh T, Gao Z, Harper SA, Shaw M (2010). Clinical aspects of pandemic 2009 influenza A (H1N1) virus infection.. N Engl J Med.

[20] Hao H, Kim TS, Braciale TJ (2008). Differential Response of Respiratory Dendritic Cell Subsets to Influenza Virus Infection.. J Virol.

[21] Grayson MH (2006). Lung dendritic cells and the inflammatory response.. Ann Allergy Asthma Immunol.

[22] Aldridge JR, Moseley CE, Boltz DA, Negovetich NJ, Reynolds C (2009). TNF/iNOS-producing dendritic cells are the necessary evil of lethal influenza virus infection.. Proc Natl Acad Sci USA.

[23] Malleret B, Manéglier B, Karlsson I, Lebon P, Nascimbeni M (2008). Primary infection with simian immunodeficiency virus: plasmacytoid dendritic cell homing to lymph nodes, type I interferon, and immune suppression.. Blood.

[24] Nascimbeni M, Perié L, Chorro L, Diocou S, Kreitmann L (2009). Plasmacytoid dendritic cells accumulate in spleens from chronically HIV-infected patients but barely participate in interferon-alpha expression.. Blood.

[25] Jiang TJ, Zhang JY, Li WG, Xie YX, Zhang XW (2010). Preferential loss of Th17 cells is associated with CD4 T cell activation in patients with 2009 pandemic H1N1 swine-origin influenza A infection.. Clin Immunol.

[26] Bermejo-Martin JF, Loeches IM, Rello J, Raquel Almansa AA (2010). Host adaptive immunity deficiency in severe pandemic influenza.. Critical Care.

[27] Arankalle VA, Lole KS, Arya RP, Tripathy AS, Ramdasi AY (2010). Role of host immune response and viral load in the differential outcome of pandemic H1N1 (2009) influenza virus infection in Indian patients.. PloS One.

